# Vets' and Pet Owners' Views About Antibiotics for Companion Animals and the Use of Phages as an Alternative

**DOI:** 10.3389/fvets.2020.513770

**Published:** 2020-09-29

**Authors:** Lucy Rhys-Davies, Jane Ogden

**Affiliations:** University of Surrey, Guildford, United Kingdom

**Keywords:** antibiotics, AMR, prescribing behaviors, companion animals, qualitative, pets, owners, veterinarian

## Abstract

Antimicrobial resistance (AMR) is a global health burden. Although a complex and multi-faceted problem, inappropriate antibiotic use has repeatedly been identified as one of the main drivers of the acceleration and spread of AMR. Behaviors associated with antibiotic prescription and use have been extensively investigated in human medicine and in the livestock sector of veterinary medicine. There is now a growing interest in the factors that drive inappropriate antibiotic use in companion animal medicine, as the significance of antimicrobial use in this sector is being recognized. Additionally, the possibility of an alternative antimicrobial, phage therapy, being implemented into companion animal medicine is explored in this study. Interviews revealed complexities in the vet-owner relationship including conflicting perceptions of responsibility regarding antibiotic prescription and use, distrust of vets' intentions, and misconception of clients' needs. A need for alternative antimicrobials was evident, as all vets were able to report difficulties finding antibiotics to treat infections as a common occurrence. Questionnaire results indicated that vets and pet owners are open to the use of phage therapy in companion animals. This study shows that an alternative antimicrobial such as phage therapy could be accepted into companion animal medicine in the UK; however, effective communication between vets and pet owners is vital if antibiotic use is to be reduced and new antimicrobials are successfully implemented.

## Introduction

Antimicrobial resistance (AMR) is a global health issue. Antibiotics underpin modern human medicine and, since the 1940s, have also been used in animals extensively, both therapeutically and non-therapeutically. Although the development of antimicrobial resistance is a natural evolutionary process for bacteria which can be mediated by a number of mechanisms, the spread of multi-resistant strains has been accelerated by the overwhelming selective pressure inflicted on bacterial populations by excessive and inappropriate use of antibiotics (1). As a result, antibiotic use has long been recognized as a significant contributor to the AMR crisis.

The One Health movement identifies the connection between human, animal, and environmental health. This approach is used by many governing bodies such as the World Health Organization (WHO) to implement legislation and inform policy-making and research, in areas where the input of multiple sectors can achieve better management of or even eliminate an issue. The effects of AMR have such a wide reach, and the contributors to the problem are so vast that identifying key areas that could successfully reduce its effects is challenging. Potential solutions to the issue require a collaborative approach across fields and disciplines, and for these reasons AMR could be considered the model “One Health” problem.

Veterinary use of antibiotics has long been recognized as an important contributor to the UK's overall antibiotic consumption, with around a third of antibiotic ingredients in the UK used in animals (2). To date, research on antibiotic use in the veterinary sector has largely focused on practice in the livestock industry where antibiotics have historically been used as growth-promoting agents and prophylactics (3). Consequently, the farming industry has long been under scrutiny amid concerns of antibiotic overuse in livestock and the impact of antibiotic residues in the environment and food chain on human health. Amid concerns suggesting the development of cross-resistance of bacteria to antibiotics used in human medicine, antibiotic growth promoters (AGPs) were banned in the EU in 1997 (3). However, the complexities of managing health and disease of livestock, as well as ensuring food safety, mean that use of antibiotics in livestock is still very high (4).

With 49% of adults owning pets (5) and high welfare standards associated with pet ownership in the UK, evidenced by the existence of multiple animal welfare charities and breeding standards (5–7), the contribution of companion animal medicine to overall antibiotic usage is of significant interest.

AMR is often thought of as a biological issue, with extensive research continuing to be carried out to battle it, from understanding mechanisms of resistance through to developing new antibiotics and alternative antimicrobials. Although the importance of biological research cannot be underestimated as treatments for multi-resistant infections are rapidly depleting, it has also been recognized that antibiotic use is also a result of human behavior. The process leading to each prescription of an antibiotic is complex and easily influenced by a variety of behavioral, environmental, and social factors. Studies in human medicine have frequently pinpointed these influences, which generally relate to the prescribing physicians' experience, perceived pressure from patients, time pressure, and decision fatigue (8). Similar studies in veterinary medicine have also highlighted factors that can contribute to antibiotic overuse, with evidence that veterinary professionals experience many of the same challenges as medical practitioners, and although the context may differ many of the burdens remain the same (9, 10).

Understanding the behavioral dynamics that can influence antibiotic use could help to predict the factors which need consideration when introducing new antimicrobials to ensure they are used appropriately, whilst also allowing for opportunities to improve antibiotic stewardship. With no new antibiotic classes discovered since the lipopeptide-derived daptomycin in 1986 (11), and the process of development including phase trials years in the making, alternative antimicrobials could play a fundamental role in reducing antibiotic use and subsequently slowing down the AMR crisis. Several alternative antimicrobials have the potential to replace antibiotics (12), including the use of bacteriophages (phages) to treat infections. Phages were discovered independently by d'Herelle and Twort in the early 1900s (13), nearly 30 years before the discovery of antibiotics. Phage therapy harnesses the natural killing ability of bacteria-specific viruses to treat infections and has been used extensively in the former Soviet Union since their discovery (13), with the Phage Therapy Center in Tbilisi, Georgia, boasting a bank of phages capable of treating a wide variety of infections (14). Lytic phages infect bacterial hosts and, like any virus, use the host cells' machinery to produce their own progeny. Following this, the phages induce lysis of the bacterial cell, killing the cell and releasing phage progeny which can then go on to infect other bacterial cells (15). Phages have also been used as tools for biocontrol of food and treatment of infections in animals (16). Although phages could offer a viable alternative to antibiotics, implementation of them into Western medicine has thus far been unsuccessful. This could be due to a lack of public awareness and acceptability, which are both paramount to ensuring a new therapy is successfully implemented.

High rates of pet ownership, high welfare standards and advanced treatments now available for pets show a huge commitment to animal health in the UK. As such, with the One Health approach and the links between human and animal health in mind, it is possible that pets could not only benefit from an alternative antimicrobial but could also be a useful tool to enable the introduction of a new antimicrobial, such as phage therapy, into the West. A growing body of work exists which explores the factors driving AMR in companion animal practice. However, to date, no research has investigated how these factors may be important in the introduction of alternative antimicrobials or explored vet and pet owners' views specifically about the use of phage therapy as an alternative to antibiotics.

## Methods

A multi-methods approach was used to explore:

- The specific social, behavioral, and circumstantial challenges that contribute to inappropriate use of antibiotics in companion animals in the UK.
- The potential uptake of an alternative antimicrobial, phage therapy, and to identify possible barriers to its implementation into companion animal medicine in the UK.

Two separate components were implemented. A qualitative component used in-depth semi-structured interviews to explore experiences of vets and pet owners in relation to antibiotic use. A quantitative component consisting of an online survey of vets and pet owners was used to identify barriers to the implementation of phage therapy into companion animal medicine. Participation was voluntary, and participants were able to withdraw up until a specified date. Written consent was provided through signed consent forms returned to the researcher via email or in person.

### Qualitative Component: Interviews With Vets and Pet Owners

This study used a qualitative design with semi-structured interviews, carried out face-to-face or on the telephone.

#### Participants and Recruitment

Some participants (*n* = 8) were recruited through the quantitative component during which they were given the option to submit their email address to be contacted for an interview regarding their experiences with antibiotics and AMR. The remaining participants were recruited by the snowballing technique and included personal and professional contacts of the researcher and veterinary partners associated with the university. Inclusion criteria for participation were as follows: (1) Vets currently working in a first-opinion companion animal practice in the UK, (2) Owners of companion animal(s) currently living in the UK.

Participants were recruited until data saturation was reached; this was determined as the point at which the data obtained from interviews did not reveal any new, significant information that influenced coding. Although there are no set guidelines for the ideal number of participants for qualitative research, studies on data saturation have shown that up to 92% of codes are often developed from between six (17) and 12 interviews (18).

#### Procedure

Telephone interviews were carried out with companion animal vets and pet owners in the UK. Participants were encouraged to speak freely about specific incidents or areas that they were interested in or felt strongly about as examples. Interviews were audio recorded and transcribed verbatim by the principal investigator. For confidentiality purposes, all interviewees were assigned numbers, and identifiable data (names, practice names, pet names) were removed from transcriptions.

Interviews with vets explored their experiences in practice, including details of their schedules, consultations and prescribing antibiotics. Vets were asked, where possible, to give specific details of cases to explain their decisions regarding treatment and experiences dealing with clients and patients, and if they would be comfortable using alternative treatments where available. Pet owners were asked to give specific details of their experiences where possible to share their knowledge of antibiotics and how they felt about managing their pets' health, and if they would be comfortable using alternative treatments where available. Interviews were guided by schedules shown in Table 1.

**Table 1:** Interview schedules for vets and pet owners.

**Vets**	**Pet owners**
• Can you talk me through a normal day of consultations? (Schedule, timings, type of ailments on a typical day) • What do you know about antibiotic resistance? • How does it affect your practice? • Can you describe a recent case where antibiotics were prescribed for a patient? (Include species and condition if possible) Can you give specific details of the case and how you came to the decision to prescribe antibiotics? • Can you describe a recent case where antibiotics could have been prescribed for a patient, but you decided not to? Can you give specific details of this case and how you came to the decision not to prescribe? • If a client wanted antibiotics for their pet but you don't want to prescribe them, would you consider using alternative treatments if they were available?	• If your pet was unwell, at what point would you decide to see the vet? Can you give examples? • Has your pet ever been unwell and not been taken to the vet? Can you give an example of a specific incident? • Can you describe an incident where your pet was prescribed antibiotics and the reasons why? • Can you describe an incident where your pet was not prescribed antibiotics and the reasons why? • Have you heard of antibiotic resistance? • Have you heard of overuse of antibiotics? • What do you think it means? • How might this influence your behavior as a pet owner? What do you think you can, or should, do? • Would you consider using alternative treatments to antibiotics if they were available?

#### Data Analysis

Thematic analysis (19) was used to identify patterns in the transcribed interviews. Analysis was carried out manually, and was an iterative, non-linear process led by the data and as detailed by Braun and Clarke (19), including several phases: Familiarization with the data through several detailed readings of the interview transcripts, generation of initial codes, searching for and reviewing themes, and defining and naming themes. Development of the themes included moving back and forth between these different phases. The first author coded the transcripts, and codes were refined through several discussions with the second author.

### Quantitative Component: Survey; Phage Therapy in Companion Animals

A short quantitative cross-sectional survey was developed using Qualtrics (Qualtrics, Provo, UT).

#### Participants and Recruitment

Participants were recruited through snowballing, which included personal and professional contacts of the researcher and veterinary partners associated with the university, and through social media platforms Twitter, LinkedIn, and Facebook where a public link to the survey was shared. Links were shared once on each platform, and participants were recruited between April and July 2017.

#### Measures

Participant demographics were measured by age, gender, education, and ethnic background.

Demographic questions were followed by a basic explanation of the principles of phage therapy. Following this, participants were asked to answer a series of questions regarding potential costs and benefits associated with the use of phage therapy.

#### Familiarity and Intentions to Use Phage Therapy

Participants were asked if they had heard of phage therapy, and if they were likely to use it to treat their pets.

#### Beliefs About Costs and Benefits of Phage Therapy

Beliefs about the costs and benefits of phage therapy were assessed using 10 items. **Benefits:** (1) It is a natural treatment, (2) Likely to be effective, (3) Could replace antibiotics, (4) Better for the environment than antibiotics, (5) Will kill resistant bacteria **Costs:** (6) Insufficient evidence for use, (7) Could lead to resistance, (8) Could cause unpleasant side effects, (9) Could be dangerous, (10) Not treating the cause of the problem. These were rated on a five-point Likert scale ranging from “Totally disagree” to “Totally agree.” For descriptive purposes only, the five-point Likert scales were reduced to: No (1, 2), Not sure (3), Yes (4, 5).

#### Analysis

SPSS statistical software V24.0 (IBM, USA) was used to analyze the results in the following ways:

- Vets' and owners' demographics, familiarity with phages, and use of phages were analyzed using descriptive statistics.
- Beliefs about the costs and benefits of phages were described using frequency statistics.
- Differences between vets' and owners' familiarity and use and beliefs about phages were defined using *t*-tests and chi-square.
- The role of beliefs about phages in predicting the intended use of phages for use in pets was assessed in vets and pet owners using multiple regression analysis.

### Ethics Statement

The multi-methods study was reviewed by and received a favorable ethical opinion from the University Ethics committee (Ref: UEC/2017/001/FHMS).

## Results

### Qualitative Component: Interviews With Vets and Pet Owners

**Figure 1:**
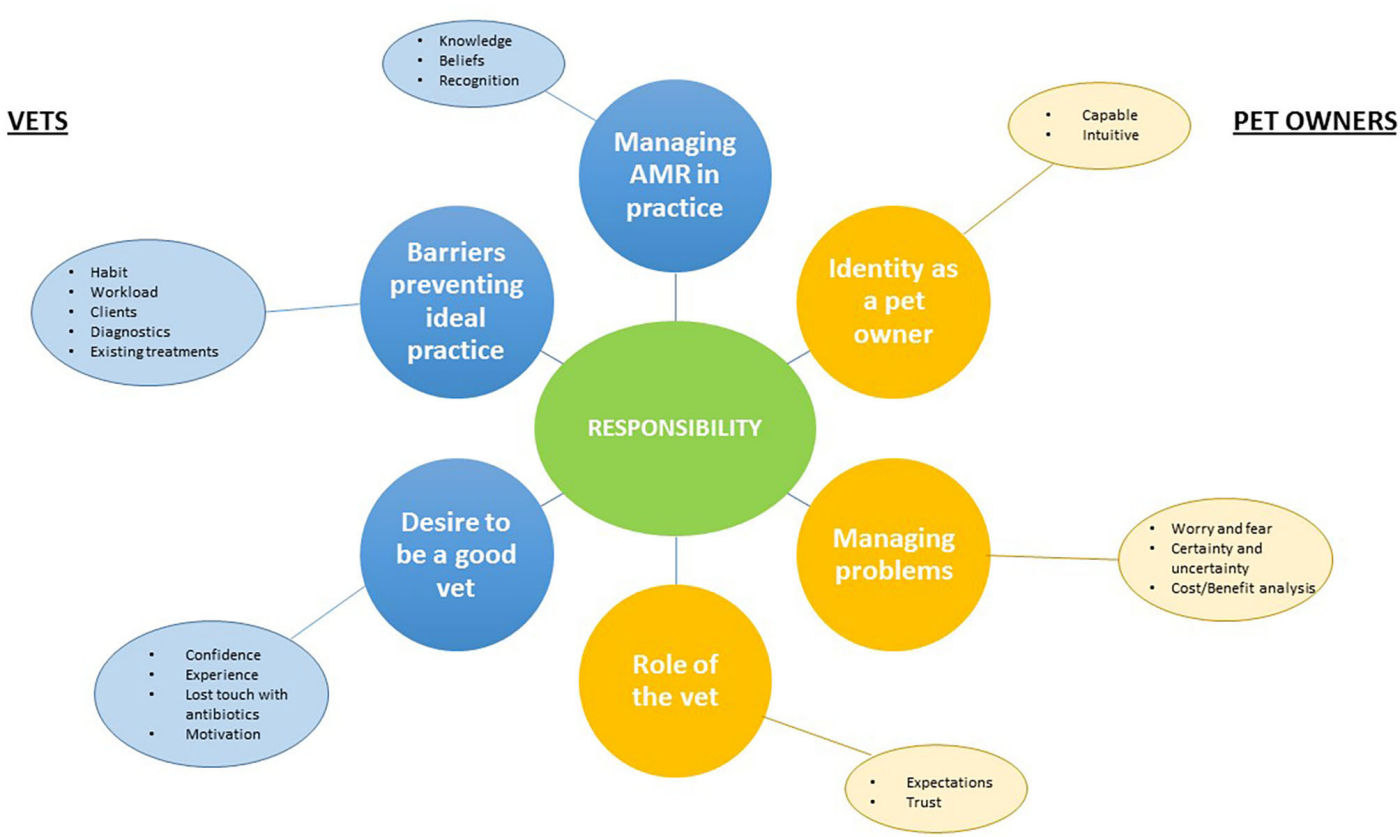
Thematic map.

Details of the participating vets and pet owners are shown in Tables 2, 3. Thematic analysis generated three key themes from the interviews with vets, three key themes from interviews with pet owners, and one additional theme which transcended both sets of interviews. Themes and sub-themes reflected by participants' experiences are shown in Table 4 and summarized in a basic thematic map (Figure 1). Quotations from vets and pet owners are shown in Tables 5, 6.

**Table 2:** Qualitative component: participant details (Vets).

**Participant**	**Age group**	**Sex**	**Years qualified**
Vet01	35–44	F	5–10
Vet02	55–64	M	11–15
Vet03	35–44	F	5–10
Vet04	25–34	M	5–10
Vet05	25–34	F	5–10
Vet06	55–64	M	20–24
Vet07	25–34	F	1–4
Vet08	25–34	F	1–4
Vet09	45–54	M	11–15
Vet10	25–34	F	1–4

**Table 3:** Qualitative component: participant details (Owners).

**Participant**	**Age group**	**Sex**	**Pets**
Owner01	45–54	F	Cats
Owner02	25–34	F	Cat, hamster
Owner03	25–34	F	Degu
Owner04	25–34	F	Dog, rabbit
Owner05	25–34	M	Dog, rabbit
Owner06	35–44	F	Dog, cat
Owner07	35–44	F	Dog, cat
Owner08	25–34	F	Dogs, chickens, guinea pigs
Owner09	25–34	F	Dog
Owner10	25–34	F	Dog, horse

**Table 4:** Qualitative component: table of themes derived from interviews.

**Vets**	**Pet owners**
**1. Management of AMR in practice** • Knowledge • Beliefs • Recognition of AMR as a problem	**1. Identity as a pet owner** • Good pet owner • Intuition
**2. Barriers preventing ideal practice** • Habit • Workload • Clients • Diagnostics • Existing treatments	**2. Managing problems** • Worry and fear • Certainty and uncertainty • Cost/Benefit analysis
**3. Desire to be a good vet** • Confidence • Experience • Lost touch with antibiotics • Motivated	**3. Role of the vet** • Expectation: Make pet better • Trust in vet • Suspicion of vet
**Transcending theme: Responsibility**	
• Of the vet	
• Of the pet owner	

**Table 5:** Quotations from Vet interviews.

**1. Managing antibiotic resistance in practice**	
Knowledge	“If it wasn't too inflamed we'd be managing it with a chlorhexidine-based wash to try and keep it under control with anti-inflammatories without throwing antibiotics at it unnecessarily.” (Vet 01)
Beliefs	“For me the key thing is that we don't use it [antibiotics] unless we feel we have to, and when we do, we follow guidelines on what's considered to be first-line usage and we only escalate onto second-line, third-line antibiotics on the back of culture and sensitivities, so I think increasingly we're… I'd like to think we're fairly good here…” (Vet 04)
Recognition of AMR as a problem	“We're seeing an increase in resistance of individual infections to what we would call the normal antibiotics that we are used to reaching for on our shelves…” (Vet 03)
**2. Barriers preventing ideal practice**	
Habit	“I knew I could treat symptomatically so I could pick my favorite antibiotic.” (Vet 01)
Workload	“I get a lot of, maybe tricky ones sort of passed on to me so vaguely unwell animals I see quite a lot of, and I tend to get quite a lot of skin cases, and um, yeah, cardiac cases as well, cardio-respiratory I tend to get referred onto me.” (Vet 04)
Clients	“People have very high expectations of vets because they can get an appointment the same day… they then expect us to fix their animals same day, and sometimes you just have to manage the owner expectations.” (Vet 07)
Diagnostics	“…sometimes financial constraints are a bit of a hassle for us in general practice as you would like to base everything on a culture and sensitivity but it's just not possible to spend that extra 60 quid.” (Vet 05)
Existing treatments	“I feel that there's a lot of polypharmacy going on, you know most of them will have 3, 3 ingredients, an anti-inflammatory, an anti-fungal and anti-bacterial whereas a lot of the time you've just got yeast in there so you want an anti-fungal.” (Vet 05)
**3. Desire to be a good vet**	
Experience	“I found that by standard I would never prescribe less than a 5 day course, and generally it will be more than 7 days… but I always find it difficult to kind of know when to stop the antibiotics. So I just kind of try to get a feel for it.” (Vet 08)
Confidence	“…now that I'm older it's like “no” and I don't care, I can call the practice manager if you want, you can speak to them, but we're not going to give antibiotics, and I think when I was younger, I might have caved in.” (Vet 10)
Lost touch with antibiotics	“My knowledge of antibiotics has deteriorated since qualifying in that I kind of forget which ones are sort of cell-wall inhibitors and things like that…” (Vet 01)
Motivated	“I think it's just trying to bear it in the back of your mind ‘this isn't right' and thinking back, ‘let's do better.” (Vet 04)

**Table 6:** Quotations from interviews with pet owners.

**1. IDENTITY AS PET OWNER**	
**Good pet owner**	“Well she had diarrhea for a week once, and I didn't take her [to the vet] … and I'm like oh, am I a really bad owner?” (Owner 04)
**Intuition**	“…people say “how can you tell?” but you can tell when your pet is ill, you know, your companion, you can tell when it's ill.” (Owner 01)
**2. MANAGING PROBLEMS**	
**Worry and fear**	“I don't know enough about dogs' health to take any chances.” (Owner 05)
**Certainty and uncertainty**	“I probably would give it around 24 hours to see if they perk up and then if they don't perk up, I'd probably take them to the vet and see what's going on.” (Owner 02)
**Cost/benefit analysis**	“…I didn't call a vet because I thought they're going to stitch it up and it's probably not necessary so if I can just keep it clean… I mean it was a Friday night as well so it would have been expensive.” (Owner 10)
**3. THE ROLE OF THE VET**	
**Expectation: Make pet better**	“You just want vets to wave a magic wand basically and make everything better.” (Owner 09)
**Trust**	“I probably put too much trust in the vet sometimes but they should know what they're on about.” (Owner 07)
**Suspicion**	“When they tell me ‘you need to do this' I'm very susceptible to their advice and when I get home, I'm like ‘Really?' What's actually necessary?” (Owner 10)

Responsibility

*“…I feel ultimately responsible for them, I am probably one of the guilty ones because I go charging down there like ‘fix my animal now' because I want my pet put right, because that's my responsibility.” (Owner 01)*.

*“I feel like a lot of vets also get a bit scared sometimes if things aren't working, so like the natural thing is just to be like oh I'll put it on antibiotics, even if it probably would have resolved without it. But it's like if you don't put it on antibiotics and something goes wrong, the client will always say “shouldn't you have put in on antibiotics?” I think that's my main difficulty, people expect antibiotics to fix everything.” (Vet 10)*.

#### Interviews With Companion Animal Vets

Themes from vet interviews explore the experiences of vets in primary care practice including their perception and understanding of antibiotic use and AMR, and management of problems with pets, clients, and treatment.

***Theme 1:****Managing AMR in practice*

The first theme highlights vets' experiences of AMR in their practice. Recognition of AMR as a significant problem and sufficient knowledge were indicated as important to manage cases where AMR is implicated.

Sub-themes: Recognition of AMR as a problem, Knowledge, Beliefs

Vets reported an increasing need to seek alternative treatment routes as opposed to prescribing antibiotics:

“*We're seeing an increase in resistance of individual infections to what we would call the normal antibiotics that we are used to reaching for on our shelves…”* (Vet 03)

Subsequently, many of the vets were able to give examples of seeking alternative methods to treat infections as opposed to prescribing broad-spectrum antibiotics, as described by Vet 01:

“*If it wasn't too inflamed we'd be managing it with a chlorhexidine-based wash to try and keep it under control with anti-inflammatories without throwing antibiotics at it unnecessarily.”* (Vet 01)

All of the vets reported that the practice they worked in was proactive in managing and adjusting standard protocols to deal with AMR:

“*For me the key thing is that we don't use it [antibiotics] unless we feel we have to, and when we do, we follow guidelines on what's considered to be first-line usage and we only escalate onto second-line, third-line antibiotics on the back of culture and sensitivities, so I think increasingly we're… I'd like to think we're fairly good here…”* (Vet 04)

Examples of cases where antibiotic resistance, or fear of antibiotic resistance arising, had caused complications in treatment plans were described frequently. An awareness of AMR and the implications of inappropriate use on veterinary practice was also demonstrated, with regular descriptions of adjusting treatment protocols when resistant organisms were implicated.

***Theme 2:****Barriers preventing ideal practice*

Although the vets were well aware of AMR and were able to describe cases where they had to manage treatment carefully, barriers to carrying out ideal practice—i.e., following standard treatment guidelines and protocols—were frequently described.

Sub-themes: Habit, Workload, Clients, Diagnostics, Existing treatments

Vets gave an insight into their experiences regarding antibiotic prescriptions. In particular, many of the vets described barriers that prevented them from carrying out best practice protocols:

“*…sometimes financial constraints are a bit of a hassle for us in general practice as you would like to base everything on a culture and sensitivity but it's just not possible to spend that extra 60 quid.”* (Vet 05)

Descriptions of workloads were frequently given with many vets switching between a myriad of disciplines including dermatology, cardiac and respiratory cases as well as routine health-checks in a normal day of consultations.

“*I get a lot of, maybe tricky ones sort of passed on to me so vaguely unwell animals I see quite a lot of, and I tend to get quite a lot of skin cases, and um, yeah, cardiac cases as well, cardio-respiratory I tend to get referred onto me.”* (Vet 04)

Frustration at the cost and timescale of existing diagnostic tests was expressed by some of the vets, and in some cases, trialing an antibiotic immediately was perceived by some as preferable to waiting for diagnostic results, due to fears that a patient's condition would worsen during this time if left untreated.

Additionally, the limitations of existing treatments were mentioned by several of the vets, in particular poly-pharmacy products which can contain a mixture of antibiotics, antifungals, anti-inflammatories, and steroids.

“*I feel that there's a lot of polypharmacy going on, you know most of them will have 3 ingredients, an anti-inflammatory, an anti-fungal and anti-bacterial whereas a lot of the time you've just got yeast in there so you want an anti-fungal.”* (Vet 05)

Client management was frequently reported as a challenging barrier to ideal practice, in particular the expectation that a treatment resolution would be achieved in a single consultation, and the resulting pressure to provide an immediate solution.

“*People have very high expectations of vets because they can get an appointment the same day… they then expect us to fix their animals same day, and sometimes you just have to manage the owner expectations.”* (Vet 07)

Some of the vets also indicated that habit was a factor which at times affected the way in which they made treatment decisions.

“*I knew I could treat symptomatically so I could pick my favorite antibiotic.”* (Vet 01)

***Theme 3:****Desire to be a good vet*

Vets identified their own level of experience in practice as an important factor in decision-making, particularly in relation to antibiotic prescriptions. All of vets described difficulties managing pet owners' antibiotic use, particularly when they were newly graduated or early in their careers. Some of the less experienced vets also reported a difficulty finding guidelines for antibiotic prescriptions in companion animal patients, making it problematic for a newly qualified vet to decide on the length of course appropriate for specific infections, relying instead on their colleagues for advice or learning case by case.

“*I found that by standard I would never prescribe less than a 5 day course, and generally it will be more than 7 days… but I always find it difficult to know when to stop the antibiotics. So I just kind of try to get a feel for it.”* (Vet 08)

With more experience, many of the vets described feeling confident and sure of their judgements and certain that their decisions would be backed up by the practice that they worked in, enabling them to make appropriate choices regarding antibiotic prescriptions.

“*…now that I'm older it's like “no” and I don't care, I can call the practice manager if you want, you can speak to them, but we're not going to give antibiotics, and I think when I was younger, I might have caved in.”* (Vet 10)

Some vets also reported concerns that despite their best intentions, they were not able to remember specific details about the functions of individual antibiotics.

“*My knowledge of antibiotics has deteriorated since qualifying in that I kind of forget which ones are sort of cell-wall inhibitors and things like that…”* (Vet 01)

A general concern for the potential impact of AMR on veterinary medicine was reported by all of the vets, and many described efforts they had made to ensure the best possible decisions for each individual patient. Vet 04 described how an antibiotic prescription that they weren't completely happy with would play on their mind:

“*I think it's just trying to bear it in the back of your mind ‘this isn't right' and thinking back, ‘let's do better.”'* (Vet 04)

Overall, a desire to remain motivated to treat patients appropriately, particularly with regard to antibiotic use, was frequently expressed.

#### Interviews With Pet Owners

***Theme 1:****Identity as a pet owner*
  Sub-themes: Capability, Intuition

Many of the owners expressed a strong desire to identify themselves as being good pet owners, in some cases describing how they feared being judged or being perceived as a “bad owner” for not taking their animal to the vet during illness:

“*Well she had diarrhea for a week once, and I didn't take her* [to the vet] …* and I'm like oh, am I a really bad owner?”* (Owner 04)

Owners reported that their intuition was an important factor in identifying if their pet was unwell, often leading them to act quickly to seek treatment and advice if they noticed a difference in their animal's behavior:

“*…people say “how can you tell?” but you can tell when your pet is ill, you know, your companion, you can tell when it's ill.”* (Owner 01)

Changes in character were most often cited as a reason for making the decision to visit the vet, as opposed to continuous monitoring of physical symptoms.

***Theme 2:****Managing problems*
  Sub-themes: Fear, Uncertainty, Risk perception

Most of the owners reported a degree of confidence managing their pet's health and described scenarios in which they were able to cope with treating their pet's condition without seeking veterinary intervention:

“*I probably would give it around 24 hours to see if they perk up and then if they don't perk up, I'd probably take them to the vet and see what's going on.”* (Owner 02)

Normally, this would be due to having experienced the specific condition before, or having easy access to advice and guidance from trusted sources. Contrary to this, some owners reported that their knowledge of animal health is not sufficient to manage their pet's illness alone, however mild, and as a result they would normally seek veterinary help as soon as they noticed anything unusual.

“*I don't know enough about dogs' health to take any chances.”* (Owner 05)

A cost-benefit analysis was frequently reported by owners when making the decision to seek treatment for their pet; an example given was whether to call a vet at the weekend, a decision in which the financial implications of calling a vet out-of-hours would have to be carefully considered.

“*…I didn't call a vet because I thought they're going to stitch it up and it's probably not necessary so if I can just keep it clean… I mean it was a Friday night as well so it would have been expensive.”* (Owner 10)

Owners identified some of the thought processes involved in making the decision to visit the vet. The point at which they would decide to take their pet to see a vet varied largely depended on fear their pet's well-being, understanding of possible treatments that could be provided, and their own confidence managing their pet's health.

***Theme 3:****The role of the vet*
  Sub-themes: Expectations, Trust, Suspicion

Owners reported conflicting perceptions of their vet; most reported trusting their vet's decisions and as a result held high expectations of the vet's ability to resolve their pet's condition.

“*You just want vets to wave a magic wand basically and make everything better.”* (Owner 09)

Owners also indicated that they felt conscious of how much they trusted their vet to make decisions regarding their pet's welfare:

“*I probably put too much trust in the vet sometimes but they should know what they're on about.”* (Owner 07)

However, some of the owners also voiced a suspicion regarding their vet's motivations to prescribe certain treatments, including antibiotics, with several stating that their vet over-prescribes.

“*When they tell me ‘you need to do this' I'm very susceptible to their advice and when I get home, I'm like ‘Really?' What's actually necessary?”* (Owner 10)

Interviews highlighted that pet owners have high expectations of vets' abilities to treat their pets; however, suspicions with regard to their motivations also exist in some cases.

***Transcending theme:*** *Responsibility*
  Sub-themes: For the pet, For the prescription

Although perceptions differed between vets and owners' experiences of clinical consultations, transcending both sets of interviews was the common theme of responsibility.

Pet owners reported feeling and accepting overall responsibility for their pets' every day care. Part of this role would include the decision to consult a vet, although the point at which owners felt this was necessary varied between individuals.

“*…I feel ultimately responsible for them, I am probably one of the guilty ones because I go charging down there like ‘fix my animal now' because I want my pet put right, because that's my responsibility.”* (Owner 01)

The feeling of responsibility in vets was expressed in relation to actual treatment decisions, such as using antibiotics correctly, specifically prescribing appropriately:

“*…how will I live with myself giving that [antibiotic] when I know so much about what's happening in humans.”* (Vet 10).

### Quantitative Component: Survey—Phage Therapy in Companion Animals

#### Participant Demographics

A total of 157 participants took the survey, consisting of 109 pet owners and 48 companion animal vets. Vets' and owners' demographics are shown in Table 7.

**Table 7:** Demographic data for Vets and pet owners (Quantitative component).

	**Vet**	**Owner**	**Total**
Gender	*N* (%)	*N* (%)	*N* (%)
Male	13 (27%)	11 (10%)	24 (15%)
Female	35 (73%)	98 (90%)	133 (85%)
Age			
18–24	3 (6%)	17 (15%)	20 (13%)
25–34	22 (46%)	43 (40%)	65 (41%)
35–44	11 (23%)	20 (18%)	31 (20%)
45+	12 (25%)	29 (27%)	41 (26%)
Education			
No formal qualifications	0	1 (1%)	1 (1%)
GCSE or equivalent	0	14 (13%)	14 (9%)
A Level or equivalent	1 (2%)	20 (18%)	21 (13%)
Undergraduate degree	29 (60%)	41 (38%)	70 (45%)
Post-graduate degree	18 (38%)	33 (30%)	51 (32%)
Ethnic background			
White	45 (94%)	108 (99%)	153 (97%)
Other	3 (6%)	1 (1%)	4 (3%)

The majority of participants were female, white, and educated to at least undergraduate degree level. One vet answered as being qualified to A-Level or equivalent; however, it can be assumed that this was an error due to the need for a veterinary degree in order to practice.

#### Vets' and Owners' Familiarity With and Intended Use of Phages

Survey participants were given a short explanation of the basis of phage therapy and following this were asked if they have previously heard of phage therapy and whether they would be happy to use it to treat their pet.

Vets' and owners' familiarity and intended use of phages is shown in Table 8.

**Table 8:** Quantitative component results: Vets' and Owners' familiarity and intended use of phage therapy.

	**Vets**	**Owners**	**Differences**					
**Have you heard of phage therapy?**	*N* (%)			*N* (%)			X^2^	*P*
Yes	25 (52%)			26 (24%)			12.1	0.001
No	23 (48%)			83 (76%)				
**Would you use it for your pet?**	*N* (%)	X	SD	*N* (%)	X	SD	T	*P*
Yes	31 (65%)	0.44	0.649	80 (74%)	0.34	0.612	0.759	0.449
Not sure	13 (27%)			21 (19%)				
No	4 (8%)			8 (7%)				

More than half (52%) of the vets had heard of phage therapy and said that they would use phage therapy to treat their pet (65%). The majority of pet owners had not heard of phage therapy (76%) but said that they would use phage therapy to treat their pet (74%).

Overall results show that the majority of the participants had not heard of phage therapy (68%) but agreed that they would be happy to use it to treat their pet (71%).

There was a significant difference between vets' and owners' familiarity with phage therapy, with vets being more familiar than owners (*p* = 0.001). There was no significant difference between vets' and owners' intended use of phage therapy for pets (*p* = 0.45).

#### Vets and Owners' Beliefs About the Costs and Benefits of Using Phages

**Figure 2:**
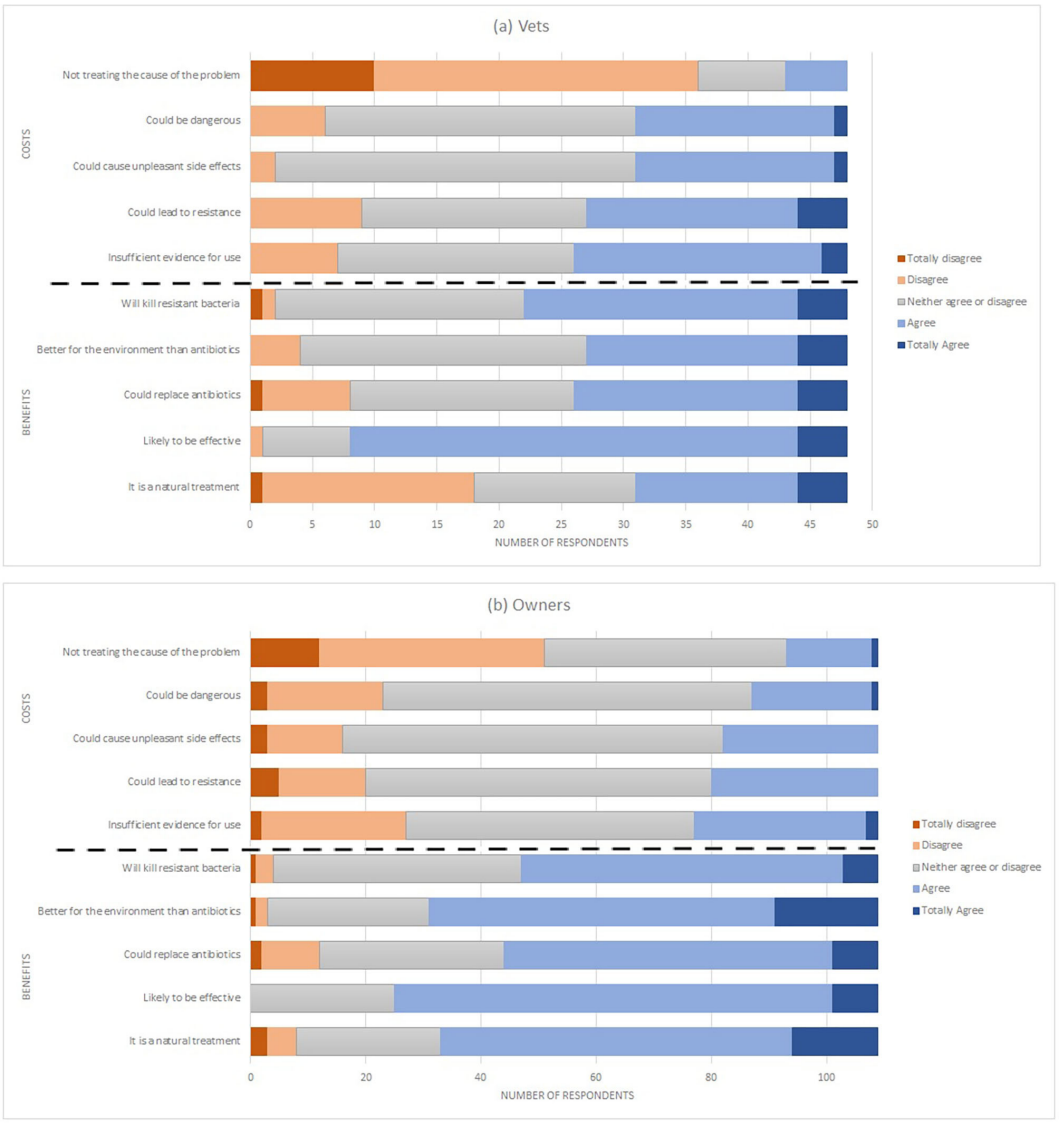
Stacked bar chart showing the breadth of responses from **(a)** Vets and **(b)** Pet Owners to questions about the costs and benefits of phage therapy.

Vets and owners' beliefs about the costs and benefits of phage therapy are shown in Figure 2 and Table 9.

**Table 9:** Quantitative component: beliefs about the costs and benefits of phage therapy.

**Benefits & Costs**	**Vets**				**Pet owners**				**Differences**	
	**Disagree**	**Not sure**	**Agree**	**Mean (SD)**	**Disagree**	**Not sure**	**Agree**	**Mean (SD)**	**t**	***P***
	***N* (%)**	***N* (%)**	***N* (%)**		***N* (%)**	***N* (%)**	***N* (%)**			
It is a natural treatment	18 (38%)	13 (27%)	17 (35%)	3.04 (1.03)	8 (7%)	25 (23%)	76 (70%)	3.73 (0.86)	−4.08	0.001
Likely to be effective	1 (2%)	7 (15%)	40 (83%)	3.9 (0.56)	0 (0%)	25 (23%)	84 (77%)	3.84 (0.53)	0.556	0.58
Could replace antibiotics	8 (17%)	18 (38%)	22 (46%)	3.35 (0.91)	12 (11%)	32 (29%)	65 (60%)	3.54 (0.83)	−1.26	0.21
Better for the environment than antibiotics	4 (8%)	23 (48%)	21 (44%)	3.44 (0.77)	3 (3%)	28 (26%)	78 (72%)	3.84 (0.75)	−3.11	0.002
Will kill resistant bacteria	2 (4%)	20 (42%)	26 (54%)	3.56 (0.77)	4 (4%)	43 (39%)	62 (57%)	3.58 (0.68)	−0.126	0.9
**Total Benefits**				3.46 (0.53)				3.71 (0.49)	−2.87	0.005
Insufficient evidence for use	7 (15%)	19 (40%)	22 (46%)	3.35 (0.79)	27 (25%)	50 (46%)	32 (29%)	3.05 (0.81)	2.22	0.028
Could lead to resistance	9 (19%)	18 (38%)	21 (44%)	3.33 (0.88)	20 (18%)	60 (55%)	29 (27%)	3.04 (0.77)	2.015	0.047
Could cause unpleasant side effects	2 (4%)	29 (60%)	17 (35%)	3.33 (0.6)	16 (15%)	66 (61%)	27 (25%)	3.07 (0.69)	2.264	0.025
Could be dangerous	6 (13%)	25 (52%)	17 (35%)	3.25 (0.7)	23 (21%)	64 (59%)	22 (20%)	2.97 (0.73)	2.231	0.027
Not treating the cause of the problem	36 (75%)	7 (15%)	5 (10%)	2.15 (0.88)	51 (47%)	42 (39%)	16 (15%)	2.58 (0.9)	−2.805	0.006
**Total Costs**				3.08 (0.5)				2.94 (0.49)	1.67	0.097

##### Costs

Vets rated the costs higher than owners in terms of: Insufficient evidence for use, Could lead to resistance, Could cause unpleasant side effects, and Could be dangerous. Owners rated the costs higher in terms of not treating the cause of the problem. The difference between total costs by vets and owners was not significant.

##### Benefits

Owners rated the benefits higher than vets in terms of being natural, better for the environment and the total benefit score. No difference was found for beliefs about effectiveness, replacing antibiotics or killing of resistant bacteria.

#### Predicting Use of Phages for Pets

The data were analyzed using multiple regression to explore the role of beliefs about costs and benefits in predicting use of phage therapy separately for vets' and pet owners.

##### Vets

For vets, beliefs about costs and benefits were used as the independent variables, and the overall model was a significant predictor of intended use of phages; predicting 19.8% of the variance (*F* = 6.7; *p* = 0.03). Results showed that intended use of phages was predicted by beliefs about benefits (*B* = 0.034; *p* = 0.02) but not by beliefs about costs (*B* = 0.27; *p* = 0.06). This indicates that greater beliefs about the benefits of phage therapy predict a greater intended use of phage therapy for pets amongst vets.

##### Owners

The second model refers to pet owners' intentions to use phage therapy for pets, and was overall a significant predictor of intended use (*F* = 14.36; *p* < 0.001), predicting 19.8% of the variance. The results show that both beliefs about benefits (*B* = 0.284; *p* = 0.002) and beliefs about costs (*B* = −0.29; *p* = 0.002) predicted intended use of phage therapy for pets. This indicates that greater beliefs about benefits and costs of phage therapy predict a greater intended use of phage therapy for pets, amongst pet owners.

## Discussion

Barriers to appropriate antibiotic use and drivers of AMR in companion animals have been explored in many recent studies (20–23), with this area of veterinary medicine of increasing interest in relation to AMR and antibiotic use. Results in this study support many findings from previous studies, in particular the point that open, adequate communication between vets and pet owners needs support to ensure appropriate antibiotic and antimicrobial use (21), and to avoid misinterpretation of intentions or motivations. Communication between vets and owners was evidently inadequate in many of the examples given both by vets and pet owners in this study and showed conflicting perceptions of responsibility regarding antibiotic prescription and use. Previous studies have recognized that the emotional bond between an owner and their pet is an important relationship which can dramatically influence subsequent behaviors, such as those related to antibiotic prescription and use (24). Other research has also shown that more than 90% of UK pet owners regard their pet as a valued family member, even to the detriment of their own health for fear of being parted from their pet (25). This bond was also evident when pet owners in this study were asked to describe their thought processes when deciding to visit a vet. Therefore, the importance of the pet–owner bond cannot be underestimated and as such should be taken into consideration when deciphering the motivations behind certain behaviors.

The findings from interviews in this study showed that owners' expectations of vets as experts and their position as prescribers could consequently mean that antibiotic prescription and use are viewed solely as the vets' responsibility, although some owners expressed concerns that their vets overprescribed or had hidden motivations other than the animal's welfare to do so. All of the vets interviewed had experienced owners pressuring them for antibiotics, or failing to administer prescribed antibiotics effectively, for example, by not finishing the course despite instructions to do so. There are many possible reasons for this which could include owners' misperceptions of vets' intentions, heightened by a suspicion that they could have ulterior motivations such as financial incentives. It could also be possible that many vets could be misreading an owner's ability to carry out treatments appropriately, although this could be difficult to ascertain as it is possible that owners may try to appear confident for fear of being seen as an incompetent or “bad” owner. This again highlights the idea that adequate communication is an integral part of ensuring appropriate antibiotic use, whether this lies with the owner to ensure they understand a prescription decision and how to administer courses of antibiotics, or with the vets to not only prescribe appropriately but to explain why antibiotics have or haven't been prescribed. Interventions or education that challenges these misperceptions by facilitating improved communication between vets and owners is likely to be an integral component in reducing unnecessary antibiotic use in companion animals.

As well as understanding behaviors surrounding use of antibiotics in order to facilitate appropriate use, the implementation of alternative antimicrobials into human and veterinary medicine could be an integral component in decreasing antibiotic use and thus reducing the incidence of untreatable multi-drug resistant (MDR) infections. However, any novel alternative antimicrobials would need careful introduction into western medicine, and subsequent communication to the general public would need to be clear to ensure adequate understanding. This process could be informed by identifying those issues which prevent appropriate antibiotic use by ascertaining the level of knowledge and awareness of AMR within companion animal medicine. With what is already known about antibiotic misuse and the factors which drive this, ensuring adequate understanding is in place when introducing alternative antimicrobials could aid to prevent or at least lessen the likelihood of misuse. Public misinformation of medicines can be extremely detrimental, an extreme example being the MMR vaccine and subsequent “anti-vax” movement which has resulted in a decline in vaccine uptake and the re-emergence of measles in the UK (26). Therefore, the importance of proper communication to ensure adequate information reaches potential consumers can't be underestimated. The possibility that pets could be useful in health literacy has been suggested also (27), and with such high rates of pet ownership in the UK alongside the global One Health movement, the success of an alternative antimicrobial in companion animal medicine could translate to acceptance and uptake into human medicine. Phage therapy has been in use since the early 1900s, before the discovery of antibiotics but, besides emergency use in extreme cases (28, 29), hasn't yet been successfully implemented into western medicine. Reasons for this could include a lack of understanding and a heavy reliance on the success of antibiotics, although it has been suggested that cultural attitudes could also be a significant factor (30). Research is ongoing exploring the use of phage therapy in food-producing animals (31, 32) and some topical phage preparations are licensed in the US for use in dogs (33), so it is reasonable to contemplate that this could be a viable alternative antimicrobial approved for use in companion animal medicine in the future.

Results from the survey showed that 75% of the participants would be happy to use phage therapy to treat a pet for an infection, after being given a brief explanation of how it works, which included the information that bacteriophages are viruses. In this study, vets were more familiar with the concept of phage therapy than pet owners were. Despite this, there was no significant difference between vets' and pet owners' willingness to use phage therapy, indicating that owners are likely to trust a vet's treatment advice even if they are unfamiliar with it themselves. In both vets and pet owners, a greater belief about the benefits of phage therapy predicted greater use of phages for treating pets, showing that with appropriate evidence, e.g., from clinical trials, validating the safety of phage therapy this alternative antimicrobial could be accepted into veterinary medicine. There are some limitations with this study, however, that need to be considered. The main issue relates to sample sizes, which were limited, and therefore has implications of generalizability and representativeness. However, the demographics of the vets were representative of the broader population of vets in the UK (34) although future research is needed to explore beliefs on a larger scale. Furthermore, beliefs about phages will evolve over time, and these results represent a snapshot of beliefs now. Future research could explore whether these beliefs change as understanding of AMR, microbes, and viruses evolves. Awareness of the potential impact of infectious diseases with limited treatment options are likely to evolve, especially considering the COVID-19 pandemic of 2020.

## Conclusions

To our knowledge, this is the first study to explore potential acceptance of phage therapy as an alternative antimicrobial in companion animal medicine in the UK.

The views of companion animal vets and pet owners on AMR and alternative antimicrobials have not yet been thoroughly investigated, and this study has given an indication of the potential challenges that could arise should alternatives be introduced, a scenario which is growing ever more likely as the need for viable treatment options increases. Results could inform more thorough investigations exploring the specific cultural and regulatory barriers preventing phage therapy from public acceptability.

## Data Availability Statement

All datasets generated for this study are included in the article/supplementary material.

## Ethics Statement

The studies involving human participants were reviewed and approved by University of Surrey Ethics Committee. The patients/participants provided their written informed consent to participate in this study.

## Author Contributions

The study was designed and the interview schedule developed by LR-D and JO. Participant recruitment was undertaken by LR-D. LR-D organized and conducted the interviews, transcribed the recorded interviews, and completed the initial coding of the transcripts. In-depth analysis and theme development was undertaken by LR-D and JO. The manuscript was written by LR-D. JO provided comments and revisions though several iterations of the manuscript. All authors contributed to the article and approved the submitted version.

## Conflict of Interest

The authors declare that the research was conducted in the absence of any commercial or financial relationships that could be construed as a potential conflict of interest.

## References

[1] Ventola CL. The antibiotic resistance crisis: part 1: causes and threats. P T. (2015) 40, 277–83. 25859123 PMC4378521

[2] Veterinary Medicines Directorate Food Standards Agency and Public Health England UK One Health Report: Antibiotic Use and Antibiotic Resistance in Animals and Humans. (2019). Available online at: https://www.gov.uk/government/publications/uk-one-health-report-antibiotic-use-and-antibiotic-resistance-in-animals-and-humans (accessed October 10, 2019).

[3] Casewell M Friis C Marco E Mcmullin P Phillips I. The European ban on growth-promoting antibiotics and emerging consequences for human and animal health. J Antimicrob Chemoth. (2003) 52:159–61. 10.1093/jac/dkg313 12837737

[4] Coyne LA Latham SM Dawson S Donald IJ Pearson RB Smith RF. Exploring perspectives on antimicrobial use in livestock: a mixed-methods study of UK pig farmers. Front Vet Sci. (2019) 6:257. 10.3389/fvets.2019.00257 31428622 PMC6688534

[5] The People's Dispensary for Sick Animals (PDSA) PDSA Animal Wellbeing (PAW) Report. (2018). Available online at: https://www.pdsa.org.uk/media/4371/paw-2018-full-web-ready.pdf (accessed October 20, 2019)

[6] National Animal Welfare Trust (NAWT) 2020 Last Update. (2020) Available online at: https://www.nawt.org.uk/ (accessed January 20, 2020)

[7] Sandøe P Corr S Palmer C Companion Animal Ethics. New York, NJ: John Wiley & Sons, Incorporated (2015).

[8] Linder JA Doctor JN Friedberg MW Reyes Nieva H Birks C Meeker D. Time of day and the decision to prescribe antibiotics. JAMA Intern Med. (2014) 174:2029–31. 10.1001/jamainternmed.2014.5225 25286067 PMC4648561

[9] Belshaw Z Robinson NJ Dean RS Brennan ML. “I Always Feel Like I Have to Rush…” pet owner and small animal veterinary surgeons' reflections on time during preventative healthcare consultations in the United Kingdom. Vet Sci. (2018) 5:20. 10.3390/vetsci5010020 29419766 PMC5876559

[10] Coyne LA Latham SM Williams NJ Dawson S Donald IJ Pearson RB. Understanding the culture of antimicrobial prescribing in agriculture: a qualitative study of UK pig veterinary surgeons. J Antimicrob Chemoth. (2016) 71:3300–12. 10.1093/jac/dkw300 27516473 PMC5079303

[11] Durand GA Raoult D Dubourg G. Antibiotic discovery: history, methods and perspectives. Int J Antimicrob Agents. (2019) 53:371–82. 10.1016/j.ijantimicag.2018.11.010 30472287

[12] Czaplewski L Bax R Clokie M Dawson M Fairhead H Fischetti VA. Alternatives to antibiotics - a pipeline portfolio review. Lancet Infect Dis. (2016) 16:239–51. 10.1016/S1473-3099(15)00466-1 26795692

[13] Summers WC. The strange history of phage therapy. Bacteriophage. (2012) 2:130–33. 10.4161/bact.20757 23050223 PMC3442826

[14] Myelnikov D. An alternative cure: the adoption and survival of bacteriophage therapy in the USSR, 1922–1955. J Hist Med All Sci. (2018) 73:385–411. 10.1093/jhmas/jry024 30312428 PMC6203130

[15] Nobrega FL Vlot M Jonge PAD Esens LL Beaumont HJE Lavigne R. Targeting mechanisms of tailed bacteriophages. Nat Rev Microbiol. (2018) 16:760–73. 10.1038/s41579-018-0070-8 30104690

[16] Tiwari R Dhama K Kumar A Rahal A Kapoor S. Bacteriophage therapy for safeguarding animal and human health: a review. Pak J Biol Sci. (2014) 17:301–15. 10.3923/pjbs.2014.301.315 24897784

[17] Mason M Sample size and saturation in PhD studies using qualitative interviews. Forum Qualitative Sozialforschung. (2010) 11:3–19. Available online at: https://www.qualitative-research.net/index.php/fqs/article/view/1428/3027

[18] Guest G Bunce A Johnson L. How many interviews are enough? Field Methods. (2006) 18:59–82. 10.1177/1525822X052799037841263

[19] Braun V Clarke V Using thematic analysis in psychology. Qual Res Psychol. (2006) 3:77–101. 10.1191/1478088706qp063oa

[20] Hardefeldt LY Gilkerson JR Billman-Jacobe H Stevenson MA Thursky K Bailey KE. Barriers to and enablers of implementing antimicrobial stewardship programs in veterinary practices. J Vet Intern Med. (2018) 32:1092–9. 10.1111/jvim.15083 29573053 PMC5980358

[21] Smith M King C Davis M Dickson A Park J Smith F. Pet owner and vet interactions: exploring the drivers of AMR. Antimicrob Resist Infect Control. (2018) 7:46–9. 10.1186/s13756-018-0341-1 29619213 PMC5879597

[22] King C Smith M Currie K Dickson A Smith F Davis M. Exploring the behavioural drivers of veterinary surgeon antibiotic prescribing: a qualitative study of companion animal veterinary surgeons in the UK. BMC Vet Res. (2018) 14:332. 10.1186/s12917-018-1646-2 30404649 PMC6223057

[23] Mateus ALP Brodbelt DC Barber N Stärk KDC. Qualitative study of factors associated with antimicrobial usage in seven small animal veterinary practices in the UK. Prev Vet Med. (2014) 117:68–78. 10.1016/j.prevetmed.2014.05.007 25091861

[24] Dickson A Smith M Smith F Park J King C Currie K Understanding the relationship between pet owners and their companion animals as a key context for antimicrobial resistance-related behaviours: an interpretative phenomenological analysis. Health Psychol Behav Med. (2019) 7:45–61. 10.1080/21642850.2019.1577738 PMC8114347 34040838

[25] McNicholas J Gilbey A Rennie A Ahmedzai S Dono J Ormerod E. Pet ownership and human health: a brief review of evidence and issues. BMJ. (2005) 331:1252–4. 10.1136/bmj.331.7527.1252 16308387 PMC1289326

[26] Wise J. MMR vaccine: Johnson urges new impetus to increase uptake as UK loses measles-free status. BMJ. (2019) 366:l5219. 10.1136/bmj.l5219 31431449

[27] Rock M Lail P. Could pets be of help in achieving health literacy? A media analysis demonstration study. Health Educ Res. (2009) 24:153–61. 10.1093/her/cyn008 18359950

[28] Lavergne S Hamilton T Biswas B Kumaraswamy M Schooley RT Wooten D. Phage therapy for a multidrug-resistant *Acinetobacter baumannii* craniectomy site infection. Open Forum Infect Dis. (2018) 5:64. 10.1093/ofid/ofy064 29687015 PMC5905571

[29] Dedrick RM Guerrero-Bustamante CA Garlena RA Russell DA Ford K Harris K. Engineered bacteriophages for treatment of a patient with a disseminated drug-resistant *Mycobacterium abscessus*. Nat Med. (2019) 25:730–3. 10.1038/s41591-019-0437-z 31068712 PMC6557439

[30] Jones EH Leterov AV Clokie M Neat science in a messy World: the global impact of human behaviour on phage therapy, past and present. Phage. (2019) 1:16–22. 10.1089/phage.2019.0002 PMC9041452 36147613

[31] Wall SK Zhang J Rostagno MH Ebner PD. Phage therapy to reduce preprocessing *Salmonella* infections in market-weight swine. Appl Environ Microbiol. (2010) 76:48–53. 10.1128/AEM.00785-09 19854929 PMC2798657

[32] Allen HK Levine UY Looft T Bandrick M Casey TA. Treatment, promotion, commotion: antibiotic alternatives in food-producing animals. Trends Microbiol. (2012) 21:114–19. 10.1016/j.tim.2012.11.001 23473629

[33] Solomon SEB Farias MRD Pimpão CT Use of *Staphylococcus aureus* phage lysate staphage lysate (SPL)® for the control of recurrent pyoderma eczema in dogs with atopic dermatitis. Acta Sci Vet. (2018) 44:7 10.22456/1679-9216.81103

[34] Vet Futures Gender Statistics About Veterinary Surgeons in the UK. (2019). Available online at: https://www.vetfutures.org.uk/download/gender-statistics-about-veterinary-surgeons-in-the-uk/ (accessed May 11, 2020)

